# Composition Analysis and Inhibitory Effect of* Sterculia lychnophora* against Biofilm Formation by* Streptococcus mutans*

**DOI:** 10.1155/2016/8163150

**Published:** 2016-04-17

**Authors:** Yang Yang, Bok-Im Park, Eun-Hee Hwang, Yong-Ouk You

**Affiliations:** ^1^Department of Food and Nutrition, School of Human Environmental Sciences, Wonkwang University, Iksan 570-749, Republic of Korea; ^2^Department of Oral Biochemistry, School of Dentistry, Wonkwang University, Iksan 570-749, Republic of Korea

## Abstract

Pangdahai is a traditional Chinese drug, specifically described in the Chinese Pharmacopoeia as the seeds of* Sterculia lychnophora* Hance. Here, we separated* S. lychnophora* husk and kernel, analyzed the nutrient contents, and investigated the inhibitory effects of* S. lychnophora* ethanol extracts on cariogenic properties of* Streptococcus mutans*, important bacteria in dental caries and plaque formation. Ethanol extracts of* S. lychnophora* showed dose-dependent antibacterial activity against* S. mutans* with significant inhibition at concentrations higher than 0.01 mg/mL compared with the control group (*p* < 0.05). Furthermore, biofilm formation was decreased by* S. lychnophora* at concentrations > 0.03 mg/mL, while bacterial viability was decreased dose-dependently at high concentrations (0.04, 0.08, 0.16, and 0.32 mg/mL). Preliminary phytochemical analysis of the ethanol extract revealed a strong presence of alkaloid, phenolics, glycosides, and peptides while the presence of steroids, terpenoids, flavonoids, and organic acids was low. The* S. lychnophora* husk had higher moisture and ash content than the kernel, while the protein and fat content of the husk were lower (*p* < 0.05) than those of the kernel. These results indicate that* S. lychnophora* may have antibacterial effects against* S. mutans*, which are likely related to the alkaloid, phenolics, glycosides, and peptides, the major components of* S. lychnophora*.

## 1. Introduction

**Figure 1:**
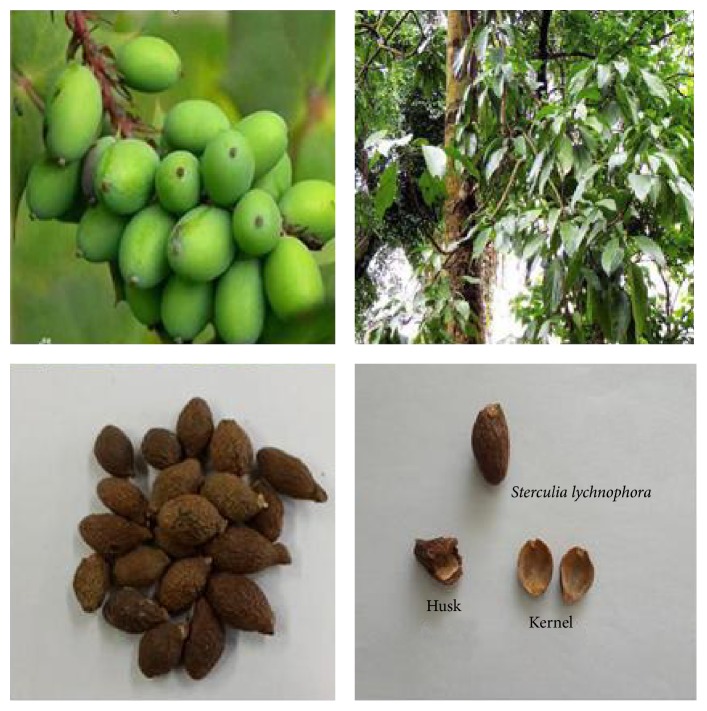
Photographs of* Sterculia lychnophora* Hance plant parts.

The Chinese traditional medicine Pangdahai consists of the dried mature seeds of* Sterculia lychnophora* Hance, a deciduous tree that belongs to the Sterculiaceae family. The trees grow mainly in tropical zones including Vietnam, India, Malaysia, Thailand, Indonesia, Gwangdong, and the Hainan Island in China [1]. The immature seed has an oval shape and looks similar to an olive while the mature seed has a length and approximate width of 2–2.5 and 1.2–1.7 cm, respectively, and weighs about 2 g (Figure 1). The outer layer of the seed is very thin and brittle, and when soaked in water its exposed interior is yellowish brown with a spongy mucus rich consistency [2].

In the dictionary of traditional Chinese medicine,* S. lychnophora* is odorless, has a viscous consistency when chewed at length, is cool or cold in nature, and has a slightly sweet or bittersweet taste. It has been used to treat the pharyngitis, constipation, and tussis in most cases [3, 4]. In China,* S. lychnophora* is commonly boiled or soaked in hot water and consumed as beverage for the treatment of sore throat or bloating. Recent report has shown neuroprotective effect [4]. However, anticariogenic effect or antibacterial activity of* S. lychnophora* is not well known. The main constituents are bassorin in the outer seed layer as well as galactose and pentose (mainly arabinose), 15.06% and 24.7%, respectively, in the peel [5].

Dental caries constitutes the most common chronic ecological disease in dentistry and is known as tooth decay or cavities. It is an infectious disease in which the hard tissues of the teeth such as the enamel, dentin, and cementum are gradually and irreversibly destroyed [6, 7]. Dental caries is caused by some types of oral* Streptococci* including the Gram-positive* Streptococcus mutans*, which is the most important cariogenic bacteria and primary causative agent of this disease [8, 9].* S. mutans* metabolizes the carbohydrates contained in consumed foods and produces organic acids, which initiate tooth enamel decay. Although fluoride compounds have been used to inhibit the formation of dental caries, high levels are cytotoxic [10]. Therefore, the development of new and safe agents that protect against the formation of dental caries is important. Natural products are good candidates for drug discovery including anticariogenic cariogenic agents. In the present study, we show* S. lychnophora* inhibits the growth as well as acid production and biofilm formation of* S. mutans*. This is the first report of anticariogenic effect of* S. lychnophora*. Furthermore, we analyzed the composition of phytochemicals and nutrient components of* S. lychnophora*.

## 2. Materials and Methods

### 2.1. Materials

Brain heart infusion (BHI) broth was purchased from Difco Laboratories (Detroit, MI, USA). Glucose and dimethyl sulfoxide (DMSO) were obtained from Sigma-Aldrich Co. (St. Louis, MO, USA).* Streptococcus mutans* ATCC 25175 was purchased from the American Type Culture Collection (ATCC, Rockville, MD, USA).

### 2.2. Plant Material and Extraction

Pangdahai was obtained from the Oriental Drug Store Dae Hak Yak Kuk (Iksan, South Korea) and was authenticated by Young-Hoi Kim at the College of Environmental and Bioresource Sciences, Chonbuk National University (Jeonju, South Korea). A voucher specimen (number 18-03-12) has been deposited at the Herbarium of the Department of Oral Biochemistry in Wonkwang University. The husk and kernels of the* S. lychnophora* plant material were separated prior to use. The ethanol extract was prepared using 300 g of plant material, which was placed in a 3000 mL flask and macerated with 3000 mL of 70% ethanol for 72 h at room temperature. The ethanol extract samples were then dried, weighed, and stored at −20°C and the yield was 10.68 g (3.56%). The ethanol extract was dissolved in DMSO to obtain the desired stock solution for the experiments. The final concentration of DMSO was adjusted to 0.1% (v/v) in the culture systems, which did not interfere with the test while control groups were treated with medium containing 0.1% DMSO.

### 2.3. Inhibition of Growth and Acid Production

Bacterial growth inhibition was determined using a modification of methods described previously [11]. The cell growth evaluation was performed at 37°C in tubes with 0.95 mL of BHI broth containing varying concentrations of* S. lychnophora* extracts. The tubes were inoculated with 0.05 mL of an overnight culture of* S. mutans* grown in BHI broth at a final density of 5 × 10^5^ colony-forming units (CFU)/mL and incubated at 37°C. After a 24 h incubation, the minimum inhibitory concentration (MIC), defined as the lowest concentration that inhibited the visible growth of* S. mutans* following overnight incubation, was determined by measuring the optical density (OD) of the growth media at 550 nm using an enzyme-linked immunosorbent assay (ELISA) plate reader (Molecular Devices Co., Sunnyvale, CA, USA). In addition, the pH of the culture media was determined using a pH meter (HANNA Instrument, Philippines). The measurements were performed in triplicate for each concentration of the test extracts and sodium fluoride (NaF, 1%) was used as a positive control.

### 2.4. Biofilm Assay

The biofilm assay used in this study was based on a method described previously [12].* S. lychnophora* extract was added to BHI broth containing 1% glucose in 35 mm polystyrene dishes or 24-well plates (Nunc, Copenhagen, Denmark). The culture media were then inoculated with a seed culture of methicillin-resistant* S. mutans* at a final density of 5 × 10^5^ CFU/mL. After culturing for 48 h at 37°C, the supernatant was removed completely, and the dishes, wells, or wells containing the composite resin teeth were rinsed with distilled water. The amount of biofilm formed in the wells was measured by staining with 0.1% safranin, followed by treatment with 30% acetic acid to release the bound safranin from the stained cells, and the absorbance of the solution was measured at 530 nm. The biofilm formed on the surface of the resin teeth was also stained with 0.1% safranin and photographed.

### 2.5. Scanning Electron Microscopy (SEM)

The biofilm formed on the 35 mm polystyrene dishes was also examined using scanning electron microscopy (SEM) using a modification of a previously described method [13]. The biofilm formed on the dishes was rinsed with distilled water and fixed with 2.5% glutaraldehyde in 0.1 M sodium cacodylate buffer (pH 7.2) at 4°C for 24 h. After sequential dehydration with graded concentrations of ethanol (60, 70, 80, 90, 95, and 100%), the samples were freeze-dried, sputter-coated with gold (108A sputter coater, Cressington Scientific Instruments Inc., Watford, England, UK), and observed using a scanning electron microscope (JSM-6360 SEM, JEOL, Tokyo, Japan).

### 2.6. Confocal Laser Scanning Microscopy

The bactericidal effect of the* S. lychnophora* extracts was determined using confocal laser scanning microscopy. The* S. mutans* culture (in BHI) was diluted with additional BHI medium to a density of approximately 1 × 10^7^ CFU/mL and then treated with high concentrations (8–64 mg/mL) of* S. lychnophora* extracts at 37°C under aerobic conditions. After a 30 min incubation, the bacteria were washed with PBS and stained using a LIVE/DEAD BacLight Bacterial Viability Kit (Molecular Probes, Eugene, OR, USA) according to the manufacturer's instructions, for 15 min. Stained bacteria were observed using a confocal laser scanning microscope (LSM 510, Zeiss, Germany). This method is based on two nucleic acid stains: green fluorescent SYTO 9 and red fluorescent propidium iodide stains, which differ in their ability to penetrate healthy bacterial cell and label live bacteria and those with damaged membranes, respectively.

### 2.7. Phytochemical Screening

The ethanol extracts of* S. lychnophora* were analyzed using phytochemical test [14]. The alkaloids, phenolics, glycosides, peptides, flavonoids, steroids, and organic acids were determined using Mayer's reagent, ferric chloride reagent, Molisch test, Biuret reagent, Mg-HCl reagent, Liebermann-Burchard reagent, and silver nitrate reagent, respectively.

### 2.8. Analytical Assays

The proximate components (moisture, protein, fat, carbohydrate, and ash) and mineral contents of the* S. lychnophora* extracts were analyzed using the Association of Official Analytical Chemists (AOAC) methods [15]. The following analyses were performed for the proximate nutrient determination. Moisture loss was determined after exposing the extract samples to a temperature of 110°C for 5 h in a forced draft oven. Total nitrogen was determined using the Kjeldahl method with a semiautomatic nitrogen analyzer. The ash content was determined by extracting ether soluble material from the extract samples with petroleum ether in a Soxhlet extractor for 8 h. Then, 2 g of the sample was charred and ashed to a constant weight at 550°C for 5 h in a muffled furnace. The carbohydrate content was calculated using the fresh weight-derived data, according to the following equation:

(1) g/100 g  carbohydrate=100 g/100 g−g/100 g  moisture+g/100 g  protein+g/100 g  fat+g/100 g  ash.

### 2.9. Mineral Content Analysis

The* S. lychnophora* extract contents of Ca, Fe, Na, K, Mg, and Zn were analyzed using an atomic absorption spectrophotometer while the P content was determined using a spectrophotometer.

### 2.10. Dietary Fiber Analysis

The* S. lychnophora* husk and kernel extracts were separately analyzed for their insoluble dietary fiber (IDF), soluble dietary fiber (SDF), and total dietary fiber (TDF) using the relevant AOAC methods [15].

### 2.11. Statistical Analysis

All the experiments were performed in triplicate and the data were analyzed using the Statistical Package for the Social Sciences (SPSS) version 18.0 program. The data are expressed as the mean ± standard error (SE). The differences between the means of the experimental and control groups were evaluated using Student's *t*-test and *p* values < 0.05 were considered statistically significant.

## 3. Results

### 3.1. Inhibition of Bacterial Growth and Acid Production

**Figure 2:**
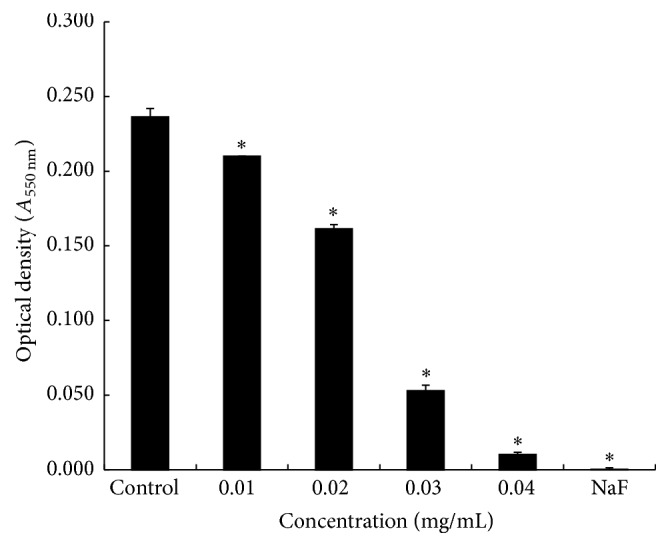
Optical density of* S. mutans* culture supernatants following treatment with varying concentrations of ethanol extracts of* S. lychnophora*. ^*∗*^ *p* < 0.05 when compared with the control group after incubation.

The ethanol extract of* S. lychnophora* showed significant dose-dependent antibacterial activity against* S. mutans* at concentrations of 0.01, 0.02, 0.03, and 0.04 mg/mL (Figure 2). Furthermore, compared to the control group, the inhibitory effects observed in the extract-treated groups were significant at concentrations higher than 0.01 mg/mL (*p* < 0.05) while the 0.1% NaF positive control exhibited antibacterial activity as well. A comparison of the extract-treated and control groups at the concentrations tested (0.01, 0.02, 0.03, and 0.04 mg/mL) revealed antibacterial effects of 11.02, 31.78, 77.54, and 95.76%, respectively.

The inhibitory effects of* S. lychnophora* ethanol extract against acid production by* S. mutans*, determined by the effects of the extract on pH, are shown in Table 1. The pH was significantly decreased in the untreated control group (pH 5.37 ± 0.01) but this effect was significantly inhibited in the positive control group (0.1% NaF, pH 7.29 ± 0.00).* S. lychnophora* extract (0.01–0.04 mg/mL) showed significant inhibition. These results indicate that the ethanol extract of* S. lychnophora* may inhibit organic acid production by* S. mutans*.

### 3.2. Inhibitory Effect of* S. lychnophora* on Biofilm Formation

**Figure 3:**
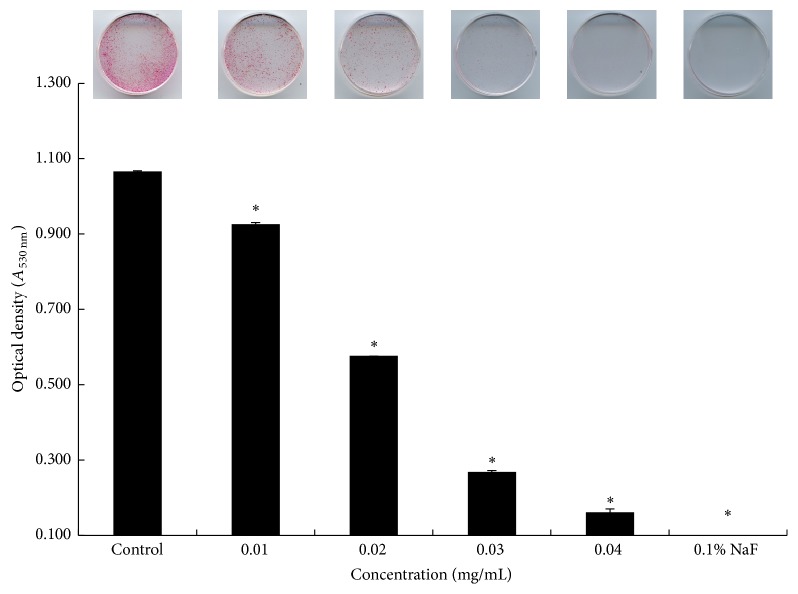
Safranin staining of* S. mutans* biofilm formation. Control, 0.01, 0.02, 0.03, and 0.04 mg/mL ethanol extract of* S. lychnophora* and positive control 0.1% sodium fluoride (NaF). ^*∗*^ *p* < 0.05 when compared with the control group after incubation.

The inhibitory effects of the extract of* S. lychnophora* on the biofilm formation by* S. mutans* evaluated using safranin staining are shown in Figure 3. The extract of* S. lychnophora* (0.01–0.04 mg/mL) inhibited the formation of* S. mutans* biofilm, which was also inhibited by the positive control (0.1% NaF). Furthermore, the* S. lychnophora* extract induced significant dose-dependent changes in the color intensity (OD) of the stained biofilm.

**Figure 4:**
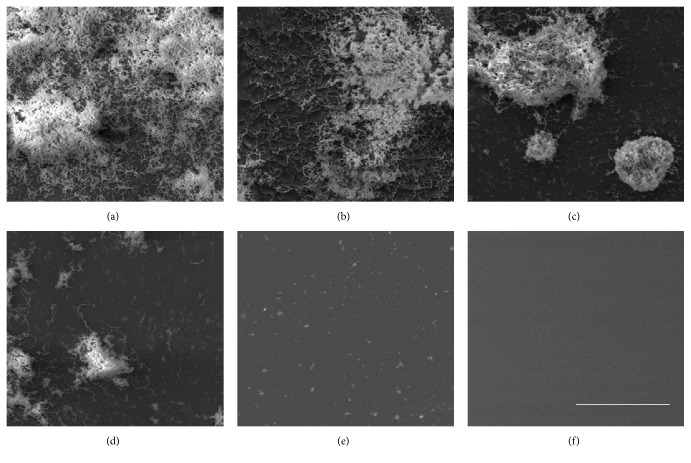
Scanning electron microscopy of* S. mutans* biofilms grown in the presence of ethanol extract of* S. lychnophora:* (a) control, (b) 0.01, (c) 0.02, (d) 0.03, and (e) 0.04 mg/mL extract and (f) positive control (0.1% NaF), scale bar = 10 *μ*m.

The SEM photographs (Figure 4) illustrate the results obtained using safranin staining.* S. mutans* attached and aggregated to the surface of the polystyrene 35 mm dishes and formed the visible biofilm in the control group. However, the biofilm formation was decreased in the presence of* S. lychnophora* at concentrations higher than 0.03 mg/mL and in the presence of the positive control (0.1% NaF).

**Figure 5:**
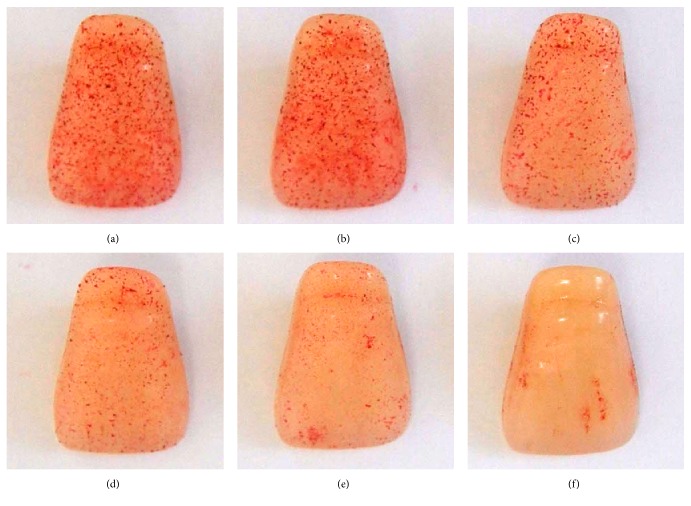
*S. mutans* biofilms on resin tooth surface following incubation with ethanol extract of* S. lychnophora:* (a) control, (b) 0.01, (c) 0.02, (d) 0.03, and (e) 0.04 mg/mL extract and (f) positive control, 0.1% sodium fluoride (NaF).

In addition, we observed biofilm formation on the surface of the resin teeth following safranin staining (Figure 5). The extract of* S. lychnophora* (0.01–0.04 mg/mL) inhibited the formation of biofilm on the surface of resin teeth, and the inhibition was particularly potent at concentrations higher than 0.03 mg/mL.

### 3.3. Bactericidal Effect of* S. lychnophora* against* S. mutans*

**Figure 6:**
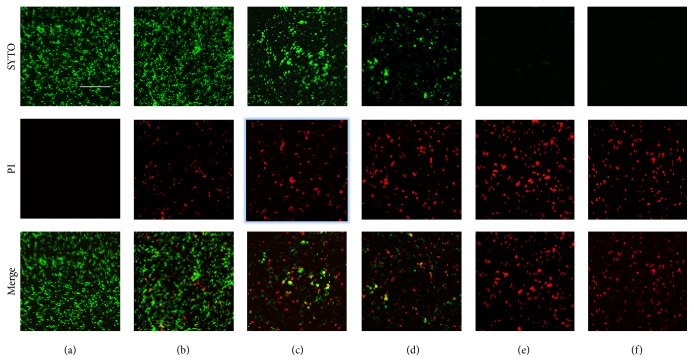
Bactericidal effect of* S. lychnophora* cultured* S. mutans* treated with* S. lychnophora* and stained with LIVE/DEAD BacLight Bacterial Viability Kit. Stained bacteria were observed using confocal laser scanning microscopy. Treatment with* S. lychnophora* decreased green-labeled living bacteria (SYTO 9 stain) and increased red-labeled dead bacteria (PI stain) dose-dependently. (a) Control, (b) 0.04, (c) 0.08, (d) 0.16, and (e) 0.32 mg/mL extract and (f) positive control sodium fluoride 0.1% (NaF), scale bar = 50 *μ*m, objective lens (×100).

The bactericidal effect of* S. lychnophora* is showed in Figure 6. The bactericidal effect of* S. lychnophora* was determined by staining the cultured bacteria with LIVE/DEAD BacLight Bacterial Viability Kit followed by confocal laser scanning microscopy. Bacterial viability decreased at high concentrations (0.04, 0.08, 0.16, and 0.32 mg/mL) of* S. lychnophora* extract, dose-dependently. This result suggests that high concentration of* S. lychnophora* extract may be bactericidal against* S. mutans*.

### 3.4. Phytochemical Screening

The results of the phytochemical tests for the ethanol extract are shown in Table 2. The preliminary phytochemical analysis is performed on the ethanol extracts. The ethanol extract revealed a strong presence of alkaloid, phenolics, glycosides, and peptides while the presence of steroids, terpenoids, flavonoids, and organic acids was low.

### 3.5. Proximate Composition

The results of the proximate composition analysis of* S. lychnophora* extracts are shown in Table 3. Moisture (11.97%  ± 0.19) and ash (5.38%  ± 0.10) of husk were higher than moisture (7.83%  ± 0.05) and ash (2.65%  ± 0.03) of kernel. Protein (3.12%  ± 0.07) and fat (0.02%  ± 0.01) of husk were lower than protein (17.24%  ± 0.23) and fat (6.47%  ± 0.25) of kernel. No significant difference was found in the levels of carbohydrate between the husk (79.54%  ± 0.21) and kernel (65.79%  ± 0.17).

### 3.6. Minerals Analysis

The results of the mineral content analysis of* S. lychnophora* extracts are shown in Table 4. The husk showed Ca, Fe, Mg, and K contents that were significantly higher (*p* < 0.05) than those of the kernel. The husk had copper levels of 0.89 ± 0.01 mg/100 g and phosphorus contents of 11.50 ± 0.01 mg/100 g, which were both significantly lower (*p* < 0.05) than those of kernel. No significant difference was found in the levels of Na between the husk and kernel.

### 3.7. Dietary Fiber Analysis

Table 5 shows the IDF, SDF, and TDF of the* S. lychnophora* husk and kernel. The IDF, SDF, and TDF of husk are higher than those of kernel significantly.

## 4. Discussion

There have been numerous research studies on the prevention and treatment of dental caries, which is one of the most frequently contracted and chronic dental diseases in humans. It is known that this disease affects 85.7% of people on average. Dental caries, once contracted, is not self-limiting and, therefore, cannot be cured without treatment [16]. Furthermore, if left untreated dental caries may develop into pulpitis, which can cause severe pain and eventually result in the necessary extraction of teeth [16].

*S. mutans* is commonly found in the dental plaque of humans and is the most cariogenic bacteria against the tooth enamel. It metabolizes dietary sugars and produces organic acids such as propionic, butyric, lactic, and formic acids as metabolic products, which can lower the pH of dental plaque and demineralize the tooth enamel and thereby initiate dental caries [17, 18].

Adhesion and colonization of* S. mutans* on the acquired enamel pellicle coated tooth surface are the initial step of the formation of dental plaque, which is a type of biofilm. The biofilm formation enhances bacterial resistance to both the host defense system and antimicrobials. Several natural substances have been developed for the treatment and prevention of dental diseases. The methanol extracts from leaves of green perilla and mugwort [19] as well as white ginseng [20] were reported to have excellent antibiotic effect against* S. mutans*. However, it was reported that ethanol extracts of* S. lychnophora* have more outstanding antibiotic effects than these substances. The methanol extracts of* Aralia continentalis* [21] as well as* Dianthus superbus* [22] were reported to have effects at 2 mg/mL and 4 mg/mL, respectively, against* S. mutans*. In addition, the ethanol extracts of* S. lychnophora* have a superior antibiotic effect to these substances as well. We aimed to provide scientific evidence of how* S. lychnophora* extracts can reduce the growth of germs that cause dental caries.

In this study, we prepared the ethanol extract of* S. lychnophora* and investigated its potential effects against the cariogenic properties of* S. mutans*. The extract of* S. lychnophora* inhibited the growth of* S. mutans*. It is commonly known as the main bacteria responsible for the formation of dental plaque and dental caries [7]. The ethanol extract of* S. lychnophora* inhibited the decrease in pH induced by* S. mutans*. These results suggest that the ethanol extract of* S. lychnophora* may inhibit organic acid production by* S. mutans*. Furthermore, the ethanol extract of* S. lychnophora* inhibited biofilm formation by* S. mutans* at concentrations ranging from 0.01 to 0.04 mg/mL and the SEM data on the biofilm formation corroborated the safranin staining data. The extract of* S. lychnophora* (0.01–0.04 mg/mL) also inhibited the biofilm formation on the resin tooth surface. In the present study, we have used 0.1% NaF as a positive control. It exhibited antibacterial activity and inhibited the decrease of pH and biofilm formation of* S. mutans* as* S. lychnophora*. However, previous reports have shown that fluoride compounds have cytotoxicity when fluoride compound was used at concentrations higher than 80 ppm [23]. Fluoride compounds have been investigated to inhibit the dental caries, but dental caries still remains the major cause of tooth loss. Therefore, it is necessary to develop new agents having better effect against dental caries. In this experiment, we found a strong presence of alkaloid, phenolics, glycosides, and peptides and a low presence of steroids, flavonoids, and organic acids. These compounds may have been responsible for the anticariogenic activity observed in the present study [24, 25].

The* S. lychnophora* ethanol extract showed levels of alkaloid, phenolics, glycosides, and peptides that were greater than those reported for the ethanol extract of* Aralia continentalis* [21]. In the results of the ethanol extract of* Aralia continentalis*, it had the strong presence of flavonoids and organic acids, moderate presence of phenolics and steroids, and the weak presence of alkaloids.

Then, we investigated the proximate composition of the* S. lychnophora* husk and kernel. The husk had moisture and ash contents that were significantly higher (*p* < 0.05) than those of the kernel as well as protein levels and fat contents that were significantly lower (*p* < 0.05) than those of kernel. According to the study by Li and Chen [26], the levels of Fe and P were 183.0 and 2786.0 mg/kg in* S. lychnophora* from China, and P was 2017.0 mg/kg in samples from Cambodia, which was more than that observed in our study. The levels of Ca, Mg, and Na at 1210.0, 3323.0, and 8.1 mg/kg, respectively, were higher in our study. The IDF level of the husk was higher than that in the peel of persimmon (15.71%), jujube (16.88%), citron (7.32%), cereal, and potato samples (7.36%) [27, 28]. The dried soymilk residue contained about 16% of SDF, which is higher than that of the husk of* S. lychnophora* [29]. The ISF level of the kernel was lower than that of the pulp of persimmon (1.95%), jujube (1.95%), and citron (2.61%) [27].

In conclusion, we demonstrated that the ethanol extract of* S. lychnophora* may inhibit acid production and biofilm formation, which may be due to the organic acids and glycosides, which are the major components of the extract of* S. lychnophora*. Therefore, we provided scientific evidence of the potential efficacy of the ethanol extract of* S. lychnophora* in the treatment of dental caries and a basis for its continued ethnomedicinal application and future development as a standard treatment.

## Figures and Tables

**Table 1:** The pH of *S. mutans* incubated with ethanol extract of *S. lychnophora*.

Conc. (mg/mL)	pH (before incubation)	pH (after incubation)
Control	7.24 ± 0.06	5.37 ± 0.01
0.01	7.26 ± 0.06	5.42 ± 0.02^*∗*^
0.02	7.25 ± 0.00	5.62 ± 0.02^*∗*^
0.03	7.25 ± 0.00	6.55 ± 0.03^*∗*^
0.04	7.25 ± 0.00	7.23 ± 0.02^*∗*^
0.1% NaF	7.30 ± 0.06	7.29 ± 0.00^*∗*^

^*∗*^ *p* < 0.05 when compared with the control group after incubation.

**Table 2:** Phytochemical screening of the ethanol extract from *S. lychnophora*.

Plant constituent	Contents
Alkaloid	+++
Phenolics	+++
Glycosides	+++
Peptides	+++
Steroids, terpenoids	+
Flavonoids	+
Organic acids	+

+++: strong; ++: moderate; +: weak; −: absent.

**Table 3:** The proximate composition of *S. lychnophora*.

	Husk (%)	Kernel (%)
Moisture	11.97 ± 0.19^*∗*^	7.83 ± 0.05^*∗*^
Protein	3.12 ± 0.07^*∗*^	17.24 ± 0.23^*∗*^
Fat	0.02 ± 0.01^*∗*^	6.47 ± 0.25^*∗*^
Ash	5.38 ± 0.10^*∗*^	2.65 ± 0.03^*∗*^
Carbohydrate	79.54 ± 0.21	65.79 ± 0.17

^*∗*^ *p* < 0.05 when compared with the husk and kernel.

**Table 4:** Mineral contents of *S. lychnophora*.

	Husk (mg/100 g)	Kernel (mg/100 g)
Calcium	367.64 ± 6.39^*∗*^	85.84 ± 3.07^*∗*^
Copper	0.89 ± 0.01^*∗*^	1.81 ± 0.08^*∗*^
Iron	6.57 ± 0.47^*∗*^	4.15 ± 0.11^*∗*^
Magnesium	405.41 ± 15.68^*∗*^	218.94 ± 5.46^*∗*^
Phosphorus	11.5 ± 0.01^*∗*^	76.81 ± 6.49^*∗*^
Potassium	1734.76 ± 33.75^*∗*^	1111.32 ± 4.77^*∗*^
Sodium	2.9 ± 0.05	2.46 ± 0.25

^*∗*^ *p* < 0.05 when compared with the husk and kernel.

**Table 5:** Content of dietary fiber in husk and kernel of *S. lychnophora*.

	Husk (mg/100 g)	Kernel (mg/100 g)
Insoluble dietary fiber (IDF)	58.08 ± 0.06^*∗*^	1.31 ± 0.01^*∗*^
Soluble dietary fiber (SDF)	14.05 ± 0.23^*∗*^	0.42 ± 0.02^*∗*^
Total dietary fiber (TDF)	72.13 ± 0.29^*∗*^	1.73 ± 0.03^*∗*^

^*∗*^ *p* < 0.05 when compared with the husk and kernel.
